# Antimicrobial resistance of commensal *Escherichia coli* and *Enterococcus faecalis* isolated from clinically healthy captive wild animals in Seoul zoo

**DOI:** 10.3389/fvets.2023.1283487

**Published:** 2024-01-11

**Authors:** Minsu Kim, Myeongsu Kim, Yong-Gu Yeo, Young-Tae Lee, Jae-Ik Han

**Affiliations:** ^1^Conservation and Health Center, Seoul Zoo, Gwacheon, Republic of Korea; ^2^Laboratory of Wildlife Medicine, College of Veterinary Medicine, Jeonbuk National University, Iksan, Republic of Korea

**Keywords:** antimicrobial resistance, zoo animals, genotype, *Escherichia coli*, *Enterococcus faecalis*

## Abstract

Despite the importance of antimicrobial resistance, only a few studies on the antimicrobial susceptibility on wild animals have been conducted owing to their population, accessibility, and characteristics. The objective of this study was to investigate the prevalence and characteristics of antimicrobial resistance pattern in *Escherichia coli* and *Enterococcus faecalis* isolated from the feces of captive wild animals in a zoo. A total of 61 captive wild animals were included in this study. *E. coli* was isolated from 58 of the 61 animals and *E. faecalis* was isolated from 29 animals. Among the isolated *E. coli* strains, ampicillin exhibited the highest resistance rate (27/29, 93.1%). Of these, 18 strains (18/29, 62%) showed multidrug resistance. The multilocus sequence typing (MLST) test showed that only ST155 was detected twice, while the other 16 strains showed different ST types. Among the *E. faecalis* strains, two were susceptible to all tested antimicrobials, whereas the remaining 27 strains showed resistance to one or more antimicrobials. Nine strains (9/27, 31%) showed multidrug resistance. Among the *E. faecalis* strains, resistance to quinupristin/dalfopristin was the highest at 96.3% (26/27), while the MLST of the nine MDR strains showed no predominant ST. Genetic association with human isolates or livestock products was observed in the isolated ST types. This indicates that antibiotic resistance in the zoo is responsible for the use of antibiotics and the partial horizontal transmission between humans and animals through feeding or contact.

## 1 Introduction

Antimicrobial resistance is recognized as a major global health problem (1). The emergence of super bacteria (e.g., methicillin-resistant *Staphylococcus aureus* [MRSA], vancomycin-resistant *S. aureus* [VRSA], vancomycin-resistant *Enterococcus* [VRE], and *Salmonella typhimurium* DT104) that are resistant to antimicrobials and the emergence of multidrug-resistant bacteria that are resistant to various antimicrobials are major global health challenges (2–4). Antimicrobial-resistant bacteria pose the 21st century’s greatest public health threat, which the world is actively combating. The emergence of antimicrobial-resistant bacteria has been systematically researched by the U.S. Food and Drug Administration (FDA) since 1996. In Korea, the National Antimicrobial Resistance Safety Management Project began in 2003. Subsequently, research to analyze the distribution status and pattern of resistant bacteria by isolating various pathogenic bacteria from humans, livestock, fish, and the environment has been earnestly promoted.

Compared with antimicrobial resistance studies on livestock or companion animals in Korea, there are few studies that address the risk of antimicrobial resistance on wild animals. This is presumed to be due to the population of wild animals, conditions of conservation facilities, and the characteristics of individual wild species. While some studies have analyzed the antimicrobial resistance rates of pathogenic *Escherichia coli* isolated from wild animals in Korea (5, 6), there have been few studies on the antimicrobial resistance of indicator bacteria isolated from wild captive animals in Korean zoos.

*Escherichia coli*, which resides as a normal bacterium in the intestines of mammals such as humans and animals, is an opportunistic bacterium that is always exposed to antimicrobials and can cause disease when immunity is weakened. *E. coli* can easily acquire and transfer antimicrobial resistance and is considered a good bioindicator for observational studies on antimicrobial resistance (7, 8). Therefore, the antimicrobial resistance of target bacteria, such as those found in the environment, meat, and companion animals, are being actively studied. *E. faecalis*, like *E. coli*, is a normal bacterium in the mammalian intestine; however, nosocomial infections in hospitals have recently emerged in humans (9). Additionally, for some antimicrobials, there are intrinsic resistances that induce resistance in bacteria regardless of the use of antimicrobials. Acquired resistance due to the misuse of antimicrobials is also possible, serving as an indicator of antimicrobial resistance (10).

Recently, zoos have tended to focus on animal welfare and species conservation (11); however, some still use methods such as petting for zoo management and increasing public interest. It is possible to become infected with zoonotic pathogens through the ingestion of animal waste through the mouth, direct contact with animals, or contaminated surfaces (12). Another concern is animal-to-human transmission of antimicrobial-resistant bacteria (13). A strong positive correlation may exist between antibiotic use and antibiotic resistance in *E. coli* (14), and several studies have reported the horizontal transmission of zoonotic diseases from zoos or petting farms (15, 16).

The objectives of this study were: (1) to investigate antimicrobial resistance and (2) to analyze the relationship between commensal *E. coli* and *E. faecalis*, which are indicator bacteria, in captive wild animals at Seoul Zoo.

## 2 Materials and methods

### 2.1 Sample collection

Samples were collected from animals that needed medical care, e.g., health checkups and anesthesia for movement, but did not show clinical symptoms. A total of 61 healthy animals belonging to 32 species were included in this study. There were 55 mammals of 27 species—including Barbary sheep (*Ammotragus lervia*) and Olive baboons (*Papio Anubis*)—5 birds of 4 species, and 1 reptile of 1 species (dwarf crocodile [*Osteolaemus tetraspis*]). Among these sampled individuals, only black-faced spoonbills (sample no. 11) and Siberian tigers (sample no. 46, 47, 48, with the same parents) were less than 1-year-old. All others were reproductive adults.

Samples were collected through rectal swabs using a sterile transport medium (Asan Pharm, Seoul, Korea) between March 2022 and September 2022. To differentiate between *E. coli* and *E. faecalis* remaining in the soil, only anal swabs were used in this study, and samples were not collected from feces. The patient information is presented in Table 1.

**Table 1:** The list of zoo animals and antimicrobial resistance included in this study.

Serial	Animals		Antimicrobial resistance^*^	
	Name	Scientific name	*E. coli*	*E. faecalis*
1	Barbary sheep	*Ammotragus lervia*	No resistance	RD-QD
2	Olive baboon	*Papio anubis*	AMP	No isolation
3	Spotted seal	*Phoca largha*	AMP-CTX-C	No isolation
4	Barbary sheep	*Ammotragus lervia*	No resistance	No isolation
5	Egyptian fruit bat	*Rousettus aegyptiacus*	F-TE	No isolation
6	Puma	*Puma concolor*	AMP-CIP	RD-QD-CIP
7	Amur leopard cat	*Prionailurus bengalensis euptilurus*	AMP-TE-DO-CIP-SXT-C-F	E-DO-TE-RD-CIP-QD
8	Amur leopard cat	*Prionailurus bengalensis euptilurus*	AMP-CTX-TE-CIP-C-SXT	E-RD-QD
9	Red fox	*Vulpes vulpes*	AMP-TE-DO	E-DO-TE-C-QD-RD-CIP
10	Black-handed spider monkey	*Ateles geoffroyi*	AMP-TE-DO	RD-QD
11	Black-faced spoonbill	*Platalea minor*	TE-DO-SXT	No isolation
12	Barbary sheep	*Ammotragus lervia*	No resistance	RD
13	Barbary sheep	*Ammotragus lervia*	No resistance	No isolation
14	Barbary sheep	*Ammotragus lervia*	No resistance	No isolation
15	Barbary sheep	*Ammotragus lervia*	No resistance	No isolation
16	Barbary sheep	*Ammotragus lervia*	No resistance	No isolation
17	North American raccoon	*Procyon lotor*	AMP-FEP-CTX-TE-DO	No resistance
18	Dwarf crocodile	*Osteolaemus tetraspis*	No isolation	No isolation
19	White stork	*Ciconia ciconia*	No isolation	RD-QD
20	Przewalski’s horse	*Equus ferus przewalskii*	AMP	No isolation
21	Western chimpanzee	*Pan troglodytes verus*	No resistance	No isolation
22	Black-faced spoonbill	*Platalea minor*	No resistance	QD
23	Spotted hyena	*Crocuta crocuta*	No isolation	QD-RD
24	Guanaco	*Lama guanicoe*	No resistance	No isolation
25	White-handed gibbon	*Hylobates lar*	AMP-TE-DO	No isolation
26	Yellow-throated marten	*Martes flavigula*	AMP-CN-SXT	QD-RD
27	Chattering lory	*Lorius garrulus*	No resistance	QD
28	Collared peccary	*Pecari tajacu*	No resistance	QD-E
29	Barbary sheep	*Ammotragus lervia*	No resistance	RD-QD
30	Barbary sheep	*Ammotragus lervia*	No resistance	No isolation
31	Barbary sheep	*Ammotragus lervia*	AMP-TE-DO-SXT-C	RD-QD
32	Barbary sheep	*Ammotragus lervia*	No resistance	No isolation
33	Barbary sheep	*Ammotragus lervia*	No resistance	No isolation
34	Barbary sheep	*Ammotragus lervia*	No resistance	No isolation
35	Barbary sheep	*Ammotragus lervia*	No resistance	No isolation
36	Indian peafowl	*Pavo cristatus*	No resistance	No isolation
37	Coyote	*Canis latrans*	No resistance	RD-QD
38	Barbary sheep	*Ammotragus lervia*	No resistance	No isolation
39	Bornean orangutan	*Pongo pygmaeus*	No resistance	No isolation
40	American black bear	*Ursus americanus*	AMP-FEP-CTX-CIP	No isolation
41	Mandrill	*Mandrillus sphinx*	No resistance	No isolation
42	Hamadryas baboon	*Papio hamadryas*	AMP-AMC-SXT	No isolation
43	Hamadryas baboon	*Papio hamadryas*	AMP-TE-DO-CIP-SXT	No isolation
44	Hamadryas baboon	*Papio hamadryas*	AMP-TE-DO-C	No isolation
45	Barbary sheep	*Ammotragus lervia*	No resistance	No isolation
46	Siberian tiger	*Panthera tigris altaica*	No resistance	E-QD-DO-TE
47	Siberian tiger	*Panthera tigris altaica*	AMP-AMC-TE-DO-CIP-C	QD
48	Siberian tiger	*Panthera tigris altaica*	No resistance	RD-QD
49	Western chimpanzee	*Pan troglodytes verus*	No resistance	RD-QD
50	Lion	*Panthera leo*	No resistance	E-DO-TE-RD-QD-CIP
51	Eurasian river otter	*Lutra lutra*	AMP-TE-SXT-C	QD
52	Red fox	*Vulpes vulpes*	AMP-FEP-CTX-CN-TE-CIP-SXT	RD-QD-CIP
53	Hamadryas baboon	*Papio hamadryas*	AMP-TE	QD
54	Hamadryas baboon	*Papio hamadryas*	AMP-AMC-SXT	No isolation
55	Banded mongoose	*Mungos mungo*	AMP-AMC-CIP	RD-QD-CIP
56	Hamadryas baboon	*Papio hamadryas*	AMP-AMC-SXT	No isolation
57	Gray wolf	*Canis lupus*	No isolation	E-AMP-QD-CIP
58	Spotted hyena	*Crocuta crocuta*	AMP-AMC-TE	No isolation
59	Barbary sheep	*Ammotragus lervia*	AMP	No isolation
60	Celebes crested macaque	*Macaca nigra*	AMP-AMC	QD-RD
61	Olive baboon	*Papio anubis*	AMP-AMC	No resistance

^*^AMP, ampicillin; AMC, amoxicillin-clavulanate; TE, tetracycline; DO, doxycycline; CIP, ciprofloxacin; CTX, cefotaxime; FEP, cefepime; CN, gentamicin; SXT, trimethoprim-sulfamethoxazole; C, chloramphenicol; E, erythromycin; RD, rifampin; QD, quinupristin-dalfopristin.

From the perspective of animal ethics, animals were not intentionally captured in this study. Captures and anal swabs were obtained only when medical care was required. Ethical clearance for this study was approved by 2019–008, 2022–004 at the Seoul Zoo IACUC. All sampling was conducted according to the committee criteria.

### 2.2 Isolation and identification of *Escherichia coli* and *Enterococcus faecalis*

The swab samples were inoculated into thioglycollate medium (BD Difco^™^, Franklin, NJ, United States) and incubated at 37°C for 24 h, and then the cultured broth was inoculated into CHROMagar^™^ *E. coli* (CHROMagar^™^, Paris, France) and CHROMagar^™^ streptococcus (CHROMagar^™^) using a sterile loop needle. Bacterial colonies selected according to the criteria for each selective medium were enriched in trypticase soy agar containing 5% sheep blood (Asan Pharm, Seoul, Korea). Species identification was performed based on the sequences of the DNA *gyrase*B gene (*E. coli*) or the 16S ribosomal RNA gene (*E. faecalis*) according to the CLSI guidelines (17). The remaining DNA extract was stored at −20°C for multilocus sequence type (MLST) analysis.

### 2.3 Antimicrobial susceptibility test

The Kirby-Bauer disk diffusion method was performed according to CLSI guidelines (18). After the turbidity was adjusted as 0.5 McFaland standard, the bacterial suspension was smeared on Muller-Hilton agar (BD BBL^™^, Franklin, NJ, United States) antimicrobial disks (Oxoid, Hampshire, United Kingdom) were placed at equal intervals, followed by incubation at 37°C for 18 h (vancomycin for 24 h). Subsequently, the size of the inhibition zone was measured, and the presence or absence of antimicrobial resistance was determined according to CLSI guidelines (18). VRE measurements might have an error in the disk diffusion method (19); therefore, they were cross-validated using the E-TEST^®^ strip (bioMerieux SA, Marcy-l’Étoile, France). For the-lactamase test, the double-disk diffusion method was used, and an amoxicillin-clavulanate disk was placed between cefepime and cefotaxime to observe diffusion. Quality control was performed using *E. coli* ATCC 25922, *S. aureus* ATCC 25923, and *E. faecalis* ATCC 29212.

While the resistance of *E. coli* was tested by disks of ampicillin (10 mcg), amoxicillin-clavulanate (30 mcg), cefepime (30 mcg), cefotaxime (30 mcg), meropenem (10 mcg), gentamicin (10 mcg), amikacin (30 mcg), sulfamethoxazole-trimethoprim (25 mcg), doxycycline (30 mcg), tetracycline (30 mcg), nitrofurantoin (300 mcg), chloramphenicol (30 mcg), and ciprofloxacin (5 mcg), the resistance of *E. faecalis* was tested by disks of penicillin G (10 U), ampicillin (10 mcg), erythromycin (15 mcg), vancomycin (30 mcg), doxycycline (30 mcg), tetracycline (30 mcg), nitrofurantoin (300 mcg), linezolid (30 mcg). chloramphenicol (30 mcg), rifampin (5 mcg), quinupristin/dalfopristin (15 mcg), and ciprofloxacin (5 mcg).

The proportion of antimicrobial-resistant strains was expressed as a percentage by dividing the number of resistant strains by the total number of positive strains. Multidrug-resistant bacteria were defined as strains showing resistance to three or more different classes of antimicrobials (20). The ratio of multi-drug resistant strains is expressed as a percentage of the total number of positive strains.

### 2.4 Multilocus sequence type

To evaluate the genetic relatedness of the isolated multidrug-resistant (MDR) bacterial clones, 7-gene MLST was conducted (Table 2). These 7 genes were amplified and sequenced to secure the nucleotide sequence of each gene, and the sequence type (ST) was determined by comparison with the PubMLST database.^1^ Based on the obtained ST type number, burst analysis was performed to analyze the genetic relationships between clones.^2^

**Table 2:** Oligonucleotide and their reaction conditions of 7-gene multilocus sequence type analysis used in this study.

Species	Locus	Primers	Sequence (5’to 3′)	Annealing temperature (°C)	Allele size (bp)	References
*Escherichia coli*	adk	ECO_adk_1F ECO_adk_1R	GCAATGCGTATCATTCTGCT CAGATCAGCGCGAACTTCAG	52	536	(42)
	fumC	ECO_FumC_1F ECO_FumC_1R	CCACCTCACTGATTCATGCG CGGTGCACAGGTAATGACTG	52	469	
	gyrB	ECO_gyrB_1F ECO_gyrB_1R	CGGGTCACTGTAAAGAAATTAT GTCCATGTAGGCGTTCAGGG	52	460	
	icd	ECO_icd_1F ECO_icd_1R	TACATTGAAGGTGATGGAATCG GTCTTTAAACGCTCCTTCGG	52	518	
	mdh	ECO_mdh_1F ECO_mdh_1R	TCTGAGCCATATCCCTACTG CGATAGATTTACGCTCTTCCA	52	452	
	purA	ECO_purA_1F ECO_purA_1R	CTGCTGTCTGAAGCATGTCC CAGTTTAGTCAGGCAGAAGC	52	478	
	recA	ECO_recA_1F ECO_RecA_1R	AGCGTGAAGGTAAAACCTGTG ACCTTTGTAGCTGTACCACG	52	510	
*Enterococcus faecalis*	gdh	EFA_gdh_1F EFA_gdh_1R	GGCGCACTAAAAGATATGGT CCAAGATTGGGCAACTTCGTCCCA	52	530	(43)
	gyd	EFA_gyd_1F EFA_gyd_1R	CAAACTGCTTAGCTCCAATGGC CATTTCGTTGTCATACCAAGC	52	395	
	pstS	EFA_pstS_1F EFA_pstS_1R	CGGAACAGGACTTTCGC ATTTACATCACGTTCTACTTGC	52	583	
	gki	EFA_gki_1F EFA_gki_1R	GATTTTGTGGGAATTGGTATGG ACCATTAAAGCAAAATGATCGC	52	438	
	aroE	EFA_aroE_1F EFA_aroE_1R	TGGAAAACTTTACGGAGACAGC GTCCTGTCCATTGTTCAAAAGC	52	459	
	xpt	EFA_xpt_1F EFA_xpt_1R	AAAATGATGGCCGTGTATTAGG AACGTCACCGTTCCTTCACTTA	52	456	
	yiqL	EFA_yiqL_1F EFA_yiqL_1R	CAGCTTAAGTCAAGTAAGTGCCG GAATATCCCTTCTGCTTGTGCT	52	436	

### 2.5 Phylogenetic analysis

To evaluate the relatedness of the isolated *E. coli* strains, neighbor-joining phylogenetic analysis for *gyr*B gene sequence was performed using MEGA X (version 10.1).

## 3 Results

### 3.1 Antimicrobial resistance ratio of isolated *Escherichia coli*

The antimicrobial resistance of the isolated *E. coli* strains is shown in Table 1. *E. coli* was isolated from 58 of the 61 animals. Although 29 strains were susceptible to all tested antimicrobials (29/58, 50%), 29 strains were resistant to more than one. The 29 strains that showed resistance to more than one antimicrobial agent were ampicillin (27/29, 93.1%), tetracycline (16/29, 55.2%), sulfamethoxazole-trimethoprim (11/29, 37.9%), doxycycline (10/29, 34.5%), ciprofloxacin (8/29, 27.6%), and amoxicillin-clavulanate (8/29, 27.6%). Conversely, no resistance to meropenem and amikacin was observed. All strains were negative in the double-disk synergy test. Of the 61 samples, 18 strains (18/58, 31%) were MDR. Among the MDR *E. coli* strains, ampicillin resistance was the highest (17/18, 94%), followed by resistance to tetracycline and sulfamethoxazole-trimethoprim. Table 3 summarizes the results for MDR *E. coli*.

**Table 3:** Antimicrobial resistance and MLST type of multidrug-resistant *E.coli* and *E. faecalis* isolated in this study.

Bacteria	Serial	Animal (scientific name)	Antimicrobial resistance^*^	ST type	Note
*Escherichia coli*	3	Spotted seal (*Phoca largha*)	AMP-CTX-C	5,415	New ST
	7	Amur leopard cat (*Prionailurus bengalensis euptilurus*)	AMP-TE-DO-CIP-SXT-C-F	164	
	8	Amur leopard cat (*Prionailurus bengalensis euptilurus*)	AMP-CTX-TE-CIP-C-SXT	2,161	New ST
	11	Black-faced spoonbill (*Platalea minor*)	TE-DO-SXT	7,224	New ST
	17	North American raccoon (*Procyon lotor*)	AMP-FEP-CTX-TE-DO	162	
	26	Yellow-throated marten (*Martes flavigula*)	AMP-CN-SXT	38	
	31	Barbary sheep (*Ammotragus lervia*)	AMP-TE-DO-SXT-C	155	
	40	American black bear (*Ursus americanus*)	AMP-FEP-CTX-CIP	349	
	42	Hamadryas baboon (*Papio hamadryas*)	AMP-AMC-SXT	206	
	43	Hamadryas baboon (*Papio hamadryas*)	AMP-TE-DO-CIP-SXT	2,448	New ST
	44	Hamadryas baboon (*Papio hamadryas*)	AMP-TE-DO-C	542	
	47	Siberian tiger (*Panthera tigris altaica*)	AMP-AMC-TE-DO-CIP-C	1994	New ST
	51	Eurasian river otter (*Lutra lutra*)	AMP-TE-SXT-C	5,173	New ST
	52	Red fox (*Vulpes vulpes*)	AMP-FEP-CTX-CN-TE-CIP-SXT	90	
	54	Hamadryas baboon (*Papio hamadryas*)	AMP-AMC-SXT	1,421	
	55	Banded mongoose (*Mungos mungo*)	AMP-AMC-CIP	7,593	
	56	Hamadryas baboon (*Papio hamadryas*)	AMP-AMC-SXT	12,646	New ST
	58	Spotted hyena (*Crocuta Crocuta*)	AMP-AMC-TE	155	
*Enterococcus faecalis*	6	Puma (*Puma concolor*)	RD-QD-CIP	721	
	7	Amur leopard cat (*Prionailurus bengalensis euptilurus*)	E-DO-TE-RD-CIP-QD	36	
	8	Amur leopard cat (*Prionailurus bengalensis euptilurus*)	E-RD-QD	116	
	9	Red fox (*Vulpes vulpes*)	E-DO-TE-C-QD-RD-CIP	116	
	46	Siberian tiger (*Panthera tigris altaica*)	E-QD-DO-TE	1,362	New ST
	50	Lion (*Panthera leo*)	E-DO-TE-RD-QD-CIP	202	
	52	Red fox (*Vulpes vulpes*)	RD-QD-CIP	32	
	55	Banded mongoose (*Mungos mungo*)	RD-QD-CIP	1,363	
	57	Gray wolf (*Canis lupus*)	E-AMP-QD-CIP	76	

^*^AMP, ampicillin; AMC, amoxicillin-clavulanate; TE, tetracycline; DO, doxycycline; CIP, ciprofloxacin; CTX, cefotaxime; FEP, cefepime; CN, gentamicin; SXT, trimethoprim-sulfamethoxazole; C, chloramphenicol; E, erythromycin; RD, rifampin; QD, quinupristin-dalfopristin.

Among the species tested, Hamadryas baboons (*Papio hamadryas*; 5/6, 80.3%) and Amur leopard cats (*Prionailurus bengalensis euptilurus*; 2/2, 100%) showed the highest MDR strain retention. Contrastingly, MDR *E. coli* was isolated from only one of the 17 barbary sheep, despite the fact that barbary sheep were the largest population tested (17/61, 27.9%). Among the 18 MDR strains, 17 species were carnivores or omnivores and only one (Barbary sheep) was an herbivore.

### 3.2 Antimicrobial resistance ratio of isolated *Enterococcus faecalis*

The antimicrobial resistance of the isolated *E. faecalis* strains is shown in Table 1. *E. faecalis* was isolated from 29 of the 61 animals. While 2 strains were susceptible to all tested antimicrobials, 27 were resistant to one or more antimicrobials. Resistance to quinipristin/dalfopristin was the highest at 96.3% (26/27), followed by rifampin at 66.7% (18/27), and ciprofloxacin at 25.9% (7/27). No resistance to penicillin, nitrofurantoin, or linezolid was observed. Of the 29 strains, 9 (31.0%) showed MDR. Among the MDR *E. faecalis*, quinupristin/dalfopristin resistance was the highest at 100% (9/9), followed by resistance to rifampin and ciprofloxacin. Compared to the non-MDR *E. faecalis* strain, an increase in ciprofloxacin resistance was observed compared to that of the non-MDR *E. faecalis* strain. All the MDR *E. faecalis* were isolated from carnivores. No vancomycin resistance *E. faecalis* strains were isolated. Table 3 summarizes the results of the MDR *E. faecalis*.

### 3.3 Multilocus sequence type

**Figure 1:**
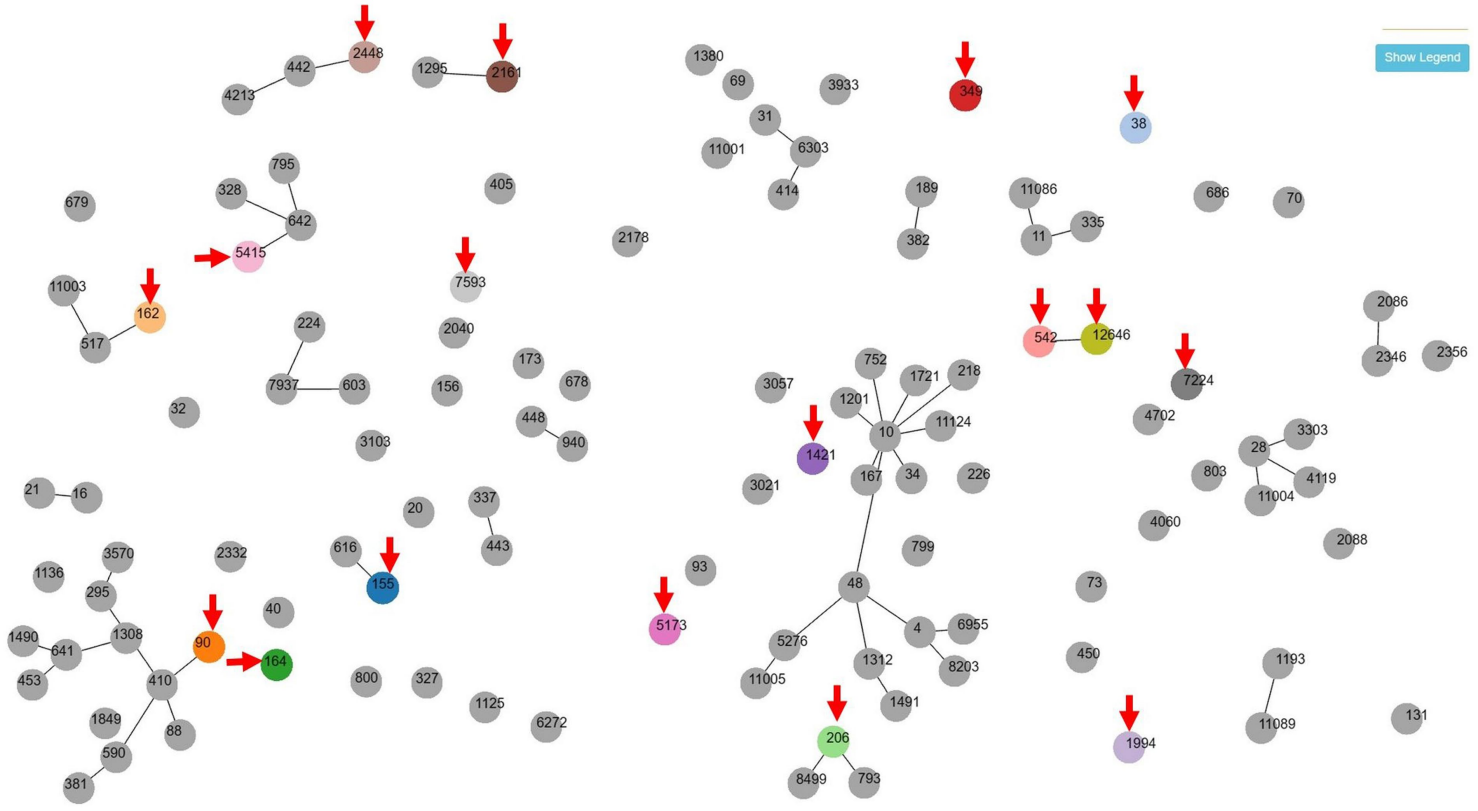
goeBURST (PHTLOViZ) analysis between multidrug-resistant *Escherichia coli* isolates of this study and strains reported in human in Korea. Seven zoo animal-origin ST types (ST90, ST155, ST162, ST206, ST2161, ST2448 and ST5415) showed single-locus variant relationships with human-origin existing Korean isolates while one ST type (ST38) was isolated in both of zoo animal (yellow-throated marten) and human. Arrows indicates the isolates of zoo animals in this study.

Multilocus sequence typing (MLST) was performed on 18 MDR strains of *E. coli* and 9 MDR strains of *E. faecalis*. Seventeen STs were identified among MDR *E. coli* (Table 3). Except for ST155, which was simultaneously detected in Barbary sheep and spotted hyena, all ST types were detected only once. Of the 17 ST types, 7 ST types (ST1994, ST2448, ST5173, ST2161, ST5415, ST7224, and ST12646) were newly discovered. The 10 previously reported ST types have been reported in various sources such as humans, livestock (dogs, cows, pigs, chickens, and turkeys), environments (river, seawater, sewage, and wastewater), wild animals (vultures, elephants, guinea fowls, and hummingbirds), and plants (spinach), suggesting wide horizontal transmission of each clone worldwide (Table 4). Compared with the ST types reported in Korea, 7 ST types (ST90, ST155, ST162, ST206, ST2161, ST2448, and ST5415) showed single-locus variants with existing Korean isolates, and all strains showing SLV relationships were isolated from humans (Figure 1). Among the 5 MDR strains isolated from hamadryas baboons, 2 showed SLV relationships with each other (ST542 and ST12646), 1 (ST206) with a human isolate, and 1 (ST2161) MDR strain of the amur leopard cat showed SLV relationships with human isolates.

**Table 4:** Information of multilocus sequence type and the clonal complex relationship of multidrug-resistant *E. coli* isolated in this study.

ST number	Animal (scientific name)	MLST							SLV* reported in Korea	
		adk	fumC	gyrB	icd	mdh	purA	recA	ST number	Origin
38	Yellow-throated marten (*Martes flavigula*)	4	26	2	25	5	5	19	None	N/A
90	Red fox (*Vulpes vulpes*)	6	4	12	1	20	8	7	410	human
155	Barbary sheep (*Ammotragus lervia*)	6	4	14	16	24	8	14	616	human
155	Spotted hyena (*Crocuta crocuta*)	6	4	14	16	24	8	14	616	human
162	North American raccoon (*Procyon lotor*)	9	65	5	1	9	13	6	517	human, environment
164	Amur leopard cat (*Prionailurus bengalensis euptilurus*)	6	4	32	16	12	8	7	None	N/A
206	Hamadryas baboon (*Papio hamadryas*)	6	7	5	1	8	18	2	793, 8,499	human
349	American black bear (*Ursus americanus*)	34	36	39	87	67	16	4	None	N/A
542	Hamadryas baboon (*Papio hamadryas*)	112	11	5	12	8	8	86	None	N/A
1,421	Hamadryas baboon (*Papio hamadryas*)	8	7	1	8	8	8	2	None	N/A
1994	Siberian tiger (*Panthera tigris altaica*)	83	14	10	14	17	94	28	None	N/A
2,161	Amur leopard cat (*Prionailurus bengalensis euptilurus*)	6	4	5	18	9	8	2	1,295	human
2,448	Hamadryas baboon (*Papio hamadryas*)	6	23	14	18	9	8	14	442	human
5,173	Eurasian river otter (*Lutra lutra*)	81	95	4	18	7	25	6	None	N/A
5,415	Spotted seal (*Phoca largha*)	9	23	64	548	11	8	6	642	human
7,224	Black-faced spoonbill (*Platalea minor*)	13	39	9	13	30	37	26	None	N/A
7,593	Banded mongoose (*Mungos mungo*)	6	29	3	18	11	26	14	None	N/A
12,646	Hamadryas baboon (*Papio hamadryas*)	112	11	5	12	8	445	86	None	N/A

*SLV, single-locus variant.

**Figure 2:**
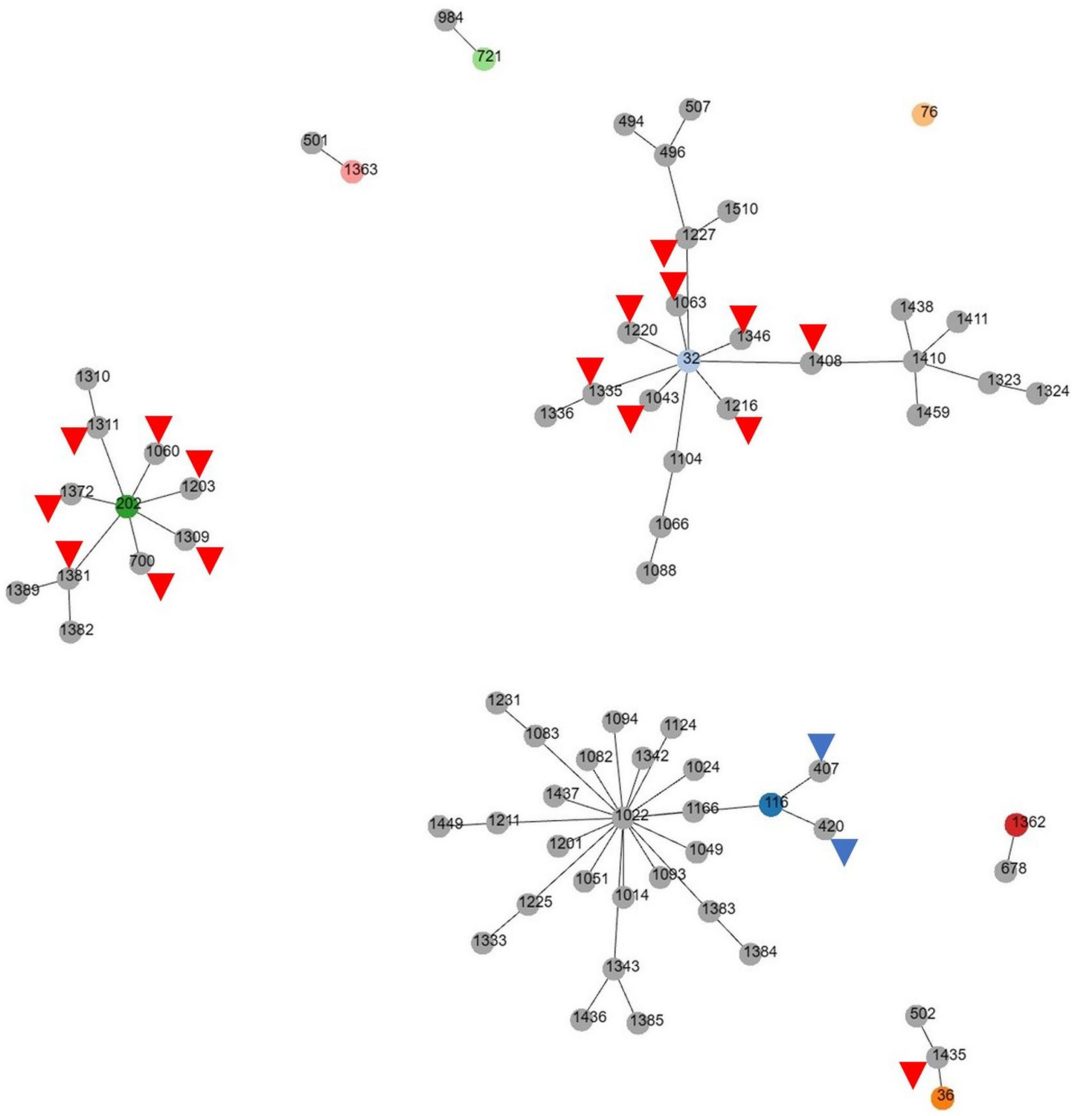
goeBURST (PHTLOViZ) analysis of multidrug-resistant *Enterococcus faecalis* reported in Korea. While three major clonal complexes containing the ST type isolated in this study are identified, ST32, ST116 and ST202 show SLV relationships with various domestic ST types isolated from pig farms (red arrowheads) or chicken (blue arrowheads).

Among the MDR *E. faecalis*, 8 ST were identified, of which 1 (ST1362) was new (Table 3). Interestingly, all but 1 of the previously reported 7 ST types (ST721, ST36, ST116, ST202, ST32, and ST 76) have been reported in humans, except for 1 (ST1363), for which the source was unknown, and 1 (ST32) out of 6 was reported from hospitalized patient specimens in China, Spain, Cuba, Germany, and Portugal. Compared to the ST type reported in Korea, three major clonal complexes containing the ST isolated in this study were identified (Figure 2). Particularly, ST32, ST36, and ST202, isolated from a red fox, amur leopard cat, and lion, respectively, showed SLV relationships with various domestic ST types isolated from pig farms (Table 5). The Puma isolate (ST721) and the spotted hyena isolate (ST984) showed close relationships at the SLV stage.

**Table 5:** Information of multilocus sequence type and the clonal complex relationship of multidrug-resistant *E. faecalis* isolated in this study.

ST number	Animal (scientific name)	MLST							SLV* reported in Korea	
		gdh	gyd	pstS	gki	aroE	xpt	yiqL	ST number	Origin
721	Puma (*Puma concolor)*	12	7	3	11	6	20	5	984	Spotted hyena
36	Amur leopard cat (*Prionailurus bengalensis euptilurus*)	16	2	19	16	17	15	11	1,435	Pig
116	Amur leopard cat (*Prionailurus bengalensis euptilurus*)	17	2	22	1	14	14	1	407, 420, 1,166	Chicken, chicken, human
116	Red Fox (*Vulpes vulpes*)	17	2	22	1	14	14	1	407, 420, 1,166	Chicken, chicken, human
1,362	Siberian tiger (*Panthera tigris altaica*)	15	4	37	1	17	1	11	678	Human
202	Lion (*Panthera leo*)	1	7	9	1	1	10	1	700, 1,060, 1,203, 1,309, 1,372	Pig
32	Red fox (*Vulpes vulpes*)	8	7	9	5	4	4	1	1,043, 1,063, 1,216, 1,220, 1,346	Pig
1,363	Banded mongoose (*Mungos mungo*)	12	7	7	5	39	2	36	501	Human
76	Gray wolf (*Canis lupus*)	22	6	7	26	22	4	4	none	

*SLV, single-locus variant.

### 3.4 Phylogenetic analysis

**Figure 3:**
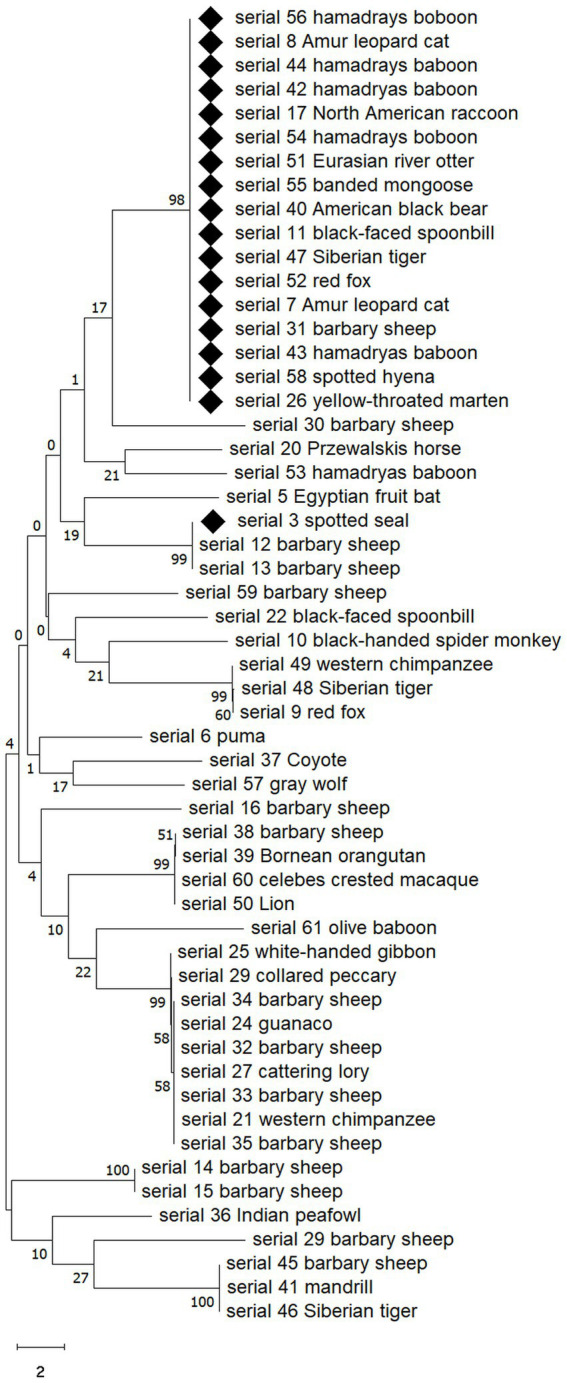
Neighbor-joining phylogenetic analysis of isolated 52 *E. coli* strains showing MDR *E. coli* appears to belong to the same clade except for one. Sequences of MDR strains are marked with diamonds. The analysis was performed by MEGA v10.0. Numbers on branches indicate bootstrap values based on 1,000 replicates.

Among 58 *E. coli* strains, a total of 52 *E. coli* strains were analyzed, including 18 MDR *E coli* strains. As a result of the analysis, the MDR strains were found to belong to the same clade except for one (ST5415; Figure 3). The 17 *E. coli* strains belonging to the same clade were composed of various species and animals with various feeding habits, showing contradictory results to MLST.

## 4 Discussion

This study was conducted to evaluate the degree and characteristics of antibiotic resistance in *E. coli* and *E. faecalis* in the intestines of clinically healthy zoo-fed wild animals. The results showed that both the isolated *E. coli* and *E. faecalis* were highly resistant to specific antibiotics (ampicillin, tetracycline, trimethoprim/sulfomethoxazole, and ciprofloxacin), besides the intrinsic resistance. Particularly, the incidence of MDR appears to be approximately 30% for both bacteria, suggesting that zoos cannot be an exception to the public health management of antibiotic resistance.

Three reasons have been suggested to explain why bacteria isolated from zoo animals acquire antibiotic resistance. First, owing to the characteristics of zoo animals, it is difficult to properly select antibiotics and administer them at an appropriate dose and duration. In zoo animals, it is often difficult to conduct appropriate tests in a timely manner when there is a need for antibiotics, and it is difficult to evaluate treatment progress. As a result, if the appropriate antibiotic is not used or if it is not used for a sufficient period and dose, it is easy for residual bacteria to acquire antibiotic resistance (21, 22). The *E. coli* isolated in this study showed high frequency of resistance to ampicillin, tetracycline, and trimethoprim/sulfamethoxazole. Owing to their wide application range and easy accessibility, these antibiotics have been widely used as empirical antibiotics in zoos, and the results of this study seem to reflect this. Among similar overseas zoo studies, a study at Chinese zoos showed that ampicillin, tetracycline, sulfamethoxazole-trimethoprim, and doxycycline have the highest *E. coli* resistance, which is similar to our results (23). However, a significant difference was that the ampicillin resistance rate in this study was 93% (27/29), which was higher than the Chinese study result (54.3%, 540/995). In the Petting Zoo of Canada, a study found that tetracycline and ampicillin resistance were the highest in captive wild animals (llamas and birds) targeting the non-O157 STEC serogroup (24). According to the results of a 2012 antimicrobial study conducted in a Japanese zoo, tetracycline, streptomycin, and ampicillin were the most common antibiotic resistance (25), which is largely consistent with the results of this study. Similar to the results of antimicrobial susceptibility studies in livestocks (26), resistance to tetracycline and ampicillin was high. The multidrug resistance rate was also higher than that of cattle (16%) but lower than that of pigs (69.7%) and chickens (82.6%) (26).

Second, food supplied to zoo animals is introduced in a state contaminated with resistant bacteria or resistant genes. The fact that antibiotic-resistant strains are more common in carnivorous and omnivorous mammals than in herbivorous mammals suggests that accumulation in the body of animals higher up in the food chain is also involved in antibiotic resistance. Among the MDR *E. coli* isolates in this study, previously reported ST types were all isolated from livestock products (chicken and cattle) and, for MDR *E. faecalis*, 4 of 7 cases were also reported from livestock products (chicken). Additionally, 17 of the 18 MDR *E. coli* strains were isolated from either carnivores or omnivores, and the fact that all *E. faecalis* isolates were isolated only from carnivores seems to reflect this. In zoos, there are cases where cheap imported food is supplied to breeding animals due to economic factors, and antibiotic resistance can be transferred through this; therefore, management measures for the current status of antibiotic resistance in the importing country or contamination of imported meat are needed.

The third factor is the potential for horizontal transmission between contact groups of animals, including zoo workers. In the case of methicillin-resistant *Staphylococcus pseudintermedius* isolated from dogs (*Canis lupus familiaris*), widespread horizontal transmission of a specific clone has been reported in the United States and Europe but not in Korea (27). A zoo is a closed and distinct ecosystem, a space in which direct contact between working veterinarians, zookeepers, and animals or indirect contact with objects, tools, and food occurs continuously, thus mutual horizontal transmission is possible. Based on the MLST and goeBurst analyses conducted in this study, 7 MDR *E. coli* isolates and three MDR *E. faecalis* isolates were found to be single-locus variants of strains reported from humans in Korea, suggesting the possibility that it is a change that occurs during the process of horizontal propagation in humans and animals. Therefore, continuous monitoring and investigation for the transmission are required to manage antibiotic resistance.

However, our results suggest that antibiotic resistance in the zoos investigated was not caused by a single factor. For example, in the case of food, carnivores receive meat provided from the same source at the same time daily; therefore, if food is the main entry route for resistance, carnivores should have the same or similar ST type, but this has not been the case. Rather, Barbary sheep and spotted hyenas with the same ST (ST155) have different diets and completely separate breeding areas, suggesting that the possibility of transmission by antibiotic use or contact with people or tools is higher than that by feeding. As a result of MLST for the MDR strain, a wide variety of ST types was detected, and only some shared the same ST, making it difficult to view horizontal transmission through direct or indirect contact between animals and humans or between animals and animals as the main route of antibiotic resistance acquisition. Resistance due to the use of antibiotics had a clear influence on the occurrence of resistance, given that resistance to antibiotics used by zoos was generally high in both strains investigated.

Of the 61 samples, Barbary sheep accounted for the majority (17 cases). The zoo investigated had about 55 Barbary sheep and regularly performed hoof care on about 20 Barbary sheep annually; therefore, the largest number of samples could be tested. All Barbary sheep ate the same feed in the same enclosure; there was little *E. coli* and *E. faecalis* resistance (5.9%, 1/17). However, as described in the results, Hamadryas baboons were the species that have highest frequency of MDR *E. coli* (83.3% 5/6). Similarly, MDR *E. faecalis* has been isolated from all carnivores, including pumas, Amur leopard cats, red foxes, Siberian tigers, lions, banded mongooses, and gray wolves. Various studies have shown that carnivores have higher multidrug resistance than omnivores or herbivores (28). Another factor that makes carnivores more resistant than other species in zoos is that, unlike herbivores, carnivores received more antimicrobials because antibiotics can be hidden in their food and can be easily administered. However, given that the biggest difference in feeding management between herbivores and carnivores is meat, the possibility that resistance factors (bacteria or gene) originate from meat cannot be ruled out (29, 30) and additional research is needed.

In this study, 7 MDR strains of *E. coli* showed SLV with human isolates in Korea while 6 MDR strains of *E. faecalis* showed SLV with human isolates in Korea. Unlike *E. coli*, where it was difficult to determine the origin of SLV because of the lack of data, it was confirmed that three ST types (ST32, ST202 and ST116) of *E. faecalis* linked several SLVs previously identified in pig (ST 32 and ST202) or chicken/human (ST116) in Korea. Interestingly, ST32 was reported to be isolated from chicken meat in Korea (31), and in this study, it was confirmed to be the branched to several ST types isolated from pigs along with ST202. On the other hand, in the case of ST116, it was confirmed that it branched from a human isolate (ST1166) and into two isolates reported in chickens (ST407 and ST420), suggesting the possibility that humans were involved in the introduction into the zoo. However, the fact that different results are obtained from the same species points out that there are various factors involved in the introduction and spread of the bacteria.

What was interesting in this study was that among the 52 isolates of *E. coli* included in the phylogenetic tree analysis, the strains that showed MDR belonged to the same clade, except for one. In particular, they belonged to the same clade regardless of animal taxon, species, or feeding habits, which was contrary to the diversity of MDR *E. coli* ST types shown in MLST. This result suggests that among *E. coli* isolates from the zoo, strains belonging to a specific clade evaluated based on the *gyr*B gene acquired MDR more easily, or that a specific strain, although the origin is unclear, gradually spread and differentiated after acquiring MDR.

Although *Enterococcus* species is a commonly found bacterium in the intestine, *E. faecalis* was isolated from only 48% (29/61) of the animals in this study. While *E. faecalis* and *E. faecium* are known to be the most abundant species in humans (32), the distribution of *Enterococcus* species in animals has been reported to vary (33). In this study, we attempted to isolate and culture *E. faecalis* from various animals raised in zoos, but no consistent pattern was observed in animal taxa, species, or type of food consumed. However, considering that *Enterococcus* species may have a relationship of exchanging resistance with each other, future studies also need to confirm the resistance patterns of major *Enterococcus* species in each animal.

This study investigated the frequency and characteristics of antibiotic-resistant bacteria in zoo wild animals kept in limited spaces. As a result of the investigation, high MDR bacterial isolation was observed in carnivores, and clones isolated from human infection sites were detected, so continuous investigation into the introduction route appears to be necessary.

## Data availability statement

The original contributions presented in the study are included in the article/supplementary material, further inquiries can be directed to the corresponding author.

## Ethics statement

The animal study was approved by Institutional Animal Care and Use Committee in Seoul zoo. The study was conducted in accordance with the local legislation and institutional requirements.

## Author contributions

MiK: Data curation, Formal analysis, Investigation, Methodology, Resources, Writing – original draft. MyK: Data curation, Investigation, Writing – original draft, Formal analysis, Software, Visualization. Y-GY: Data curation, Investigation, Writing – original draft, Resources. Y-TL: Writing – original draft, Data curation, Investigation. J-IH: Conceptualization, Formal analysis, Funding acquisition, Investigation, Methodology, Software, Supervision, Validation, Visualization, Writing – original draft, Writing – review & editing.

## Footnotes

^1^https://pubmlst.org/

^2^https://online.phyloviz.net/index
